# Robot assisted versus laparoscopic suturing learning curve in a simulated setting

**DOI:** 10.1007/s00464-019-07263-2

**Published:** 2019-11-21

**Authors:** Erik Leijte, Ivo de Blaauw, Frans Van Workum, Camiel Rosman, Sanne Botden

**Affiliations:** 1 Department of Pediatric Surgery, Radboud University Medical Centre - Amalia Children’s Hospital, Geert Grooteplein 10 Route 618, 6500HB Nijmegen, The Netherlands; 2 Department of Surgery, Radboud University Medical Centre, Nijmegen, The Netherlands

**Keywords:** Laparoscopy training, Simulation, Robotics training, Learning curve

## Abstract

**Background:**

Compared to conventional laparoscopy, robot assisted surgery is expected to have most potential in difficult areas and demanding technical skills like minimally invasive suturing. This study was performed to identify the differences in the learning curves of laparoscopic versus robot assisted suturing.

**Method:**

Novice participants performed three suturing tasks on the EoSim laparoscopic augmented reality simulator or the RobotiX robot assisted virtual reality simulator. Each participant performed an intracorporeal suturing task, a tilted plane needle transfer task and an anastomosis needle transfer task. To complete the learning curve, all tasks were repeated up to twenty repetitions or until a time plateau was reached. Clinically relevant and comparable parameters regarding time, movements and safety were recorded. Intracorporeal suturing time and cumulative sum analysis was used to compare the learning curves and phases.

**Results:**

Seventeen participants completed the learning curve laparoscopically and 30 robot assisted. Median first knot suturing time was 611 s (s) for laparoscopic versus 251 s for robot assisted (*p *< 0.001), and this was 324 s versus 165 (sixth knot, *p *< 0.001) and 257 s and 149 s (eleventh knot, *p *< 0.001) respectively on base of the found learning phases. The percentage of ‘adequate surgical knots’ was higher in the laparoscopic than in the robot assisted group. First knot: 71% versus 60%, sixth knot: 100% versus 83%, and eleventh knot: 100% versus 73%. When assessing the ‘instrument out of view’ parameter, the robot assisted group scored a median of 0% after repetition four. In the laparoscopic group, the instrument out of view increased from 3.1 to 3.9% (left) and from 3.0 to 4.1% (right) between the first and eleventh knot (*p *> 0.05).

**Conclusion:**

The learning curve of minimally invasive suturing shows a shorter task time curve using robotic assistance compared to the laparoscopic curve. However, laparoscopic outcomes show good end results with rapid outcome improvement.

**Electronic supplementary material:**

The online version of this article (10.1007/s00464-019-07263-2) contains supplementary material, which is available to authorized users.

The increasing rate in which surgical innovations are developed and implemented has placed an unprecedented pressure on surgeons to acquire technical proficiency in these new skills [1]. The learning curve is the amount of procedural training required for a surgeon to achieve competence in a new procedure. The duration of the learning curve to reach a particular outcome depends on the specific outcomes being investigated [2]. During the early phase of learning, the novice can expect to find longer task duration and poorer overall outcomes with the chance of a higher rate of complications [1, 3–5]. The skills set needed for minimally invasive surgery is very different from open surgery and with the introduction of robot assisted surgery, a new technical skill set has been added to this. Every new surgical technique and procedure has their own learning curve, and may even introduce learning associated morbidity [4]. Learning curves are expected to be completed faster using robot assisted surgery compared to laparoscopic surgery due to the possibilities of intuitive wristed movements and three dimensional vision [1]. In addition, there is no standardized method to quantify surgical skill level that resembles proficiency. The use of a single parameter, such as time to complete the task, is too simplistic since it does not take into account complications or patient related outcomes [10]. Therefore, multidimensional assessment is recommended, which incorporates the need of simulator assessment feedback with objective assessment parameters, including safety parameters, leading to a competency- or outcome-anchored appraisal of skill performance and progression [1, 11–13].

Currently only several studies have been performed to compare laparoscopic and robot assisted learning curves. In one study novices benefited from robotic assistance in terms of time, distance, smoothness and accuracy [1]. Another study which compared novices performing three sutures laparoscopically and robot assisted in a porcine model resulted in an overall improved performance of robotic assistance [6]. This was found to be statistically significant as compared to laparoscopy for the completion time, number of errors, injuries, and workload. A more recent study found novices to commit more errors during a pattern cutting task using laparoscopy compared to robot assisted participants. However, no statistically significant differences were found regarding completion time [7].

Most studies made a comparison ranging from basic tasks to intracorporeal suturing. However, robot assisted suturing has been stated to be particularly advantageous for performing complex surgical procedures, such as intracorporeal intestinal anastomoses [8, 9]. The focus of this study is therefore on these complex suturing tasks, because of potential benefit of robot assisted surgery in these complex tasks, performed by less experienced surgeons [9]. The aim of this study was to evaluate whether the learning curve of robot assisted complex suturing would be shorter and with less ‘collateral damage’ compared to conventional laparoscopic complex suturing in a simulator setting using multiple assessment parameters.

## Methods

### Participants

The study was performed at the Radboud university medical center Nijmegen, The Netherlands. Participants were eligible to participate if they had a basic understanding of the concept of minimal invasive surgery, either endoscopic or robot assisted, therefore medical interns and residents in training were recruited. Recruitment was voluntary by advertisement and group allocation was randomly based on the simulator availability and the participant’s surgical experience without stratification. Participants who already had training or clinical experience with laparoscopy but without robot assisted experience were only included in the robot assisted group. For this study no ethical approval board was required due to the non-medical setup and the voluntary participation.

### Simulators

#### EoSim laparoscopic simulator

**Fig. 1:**
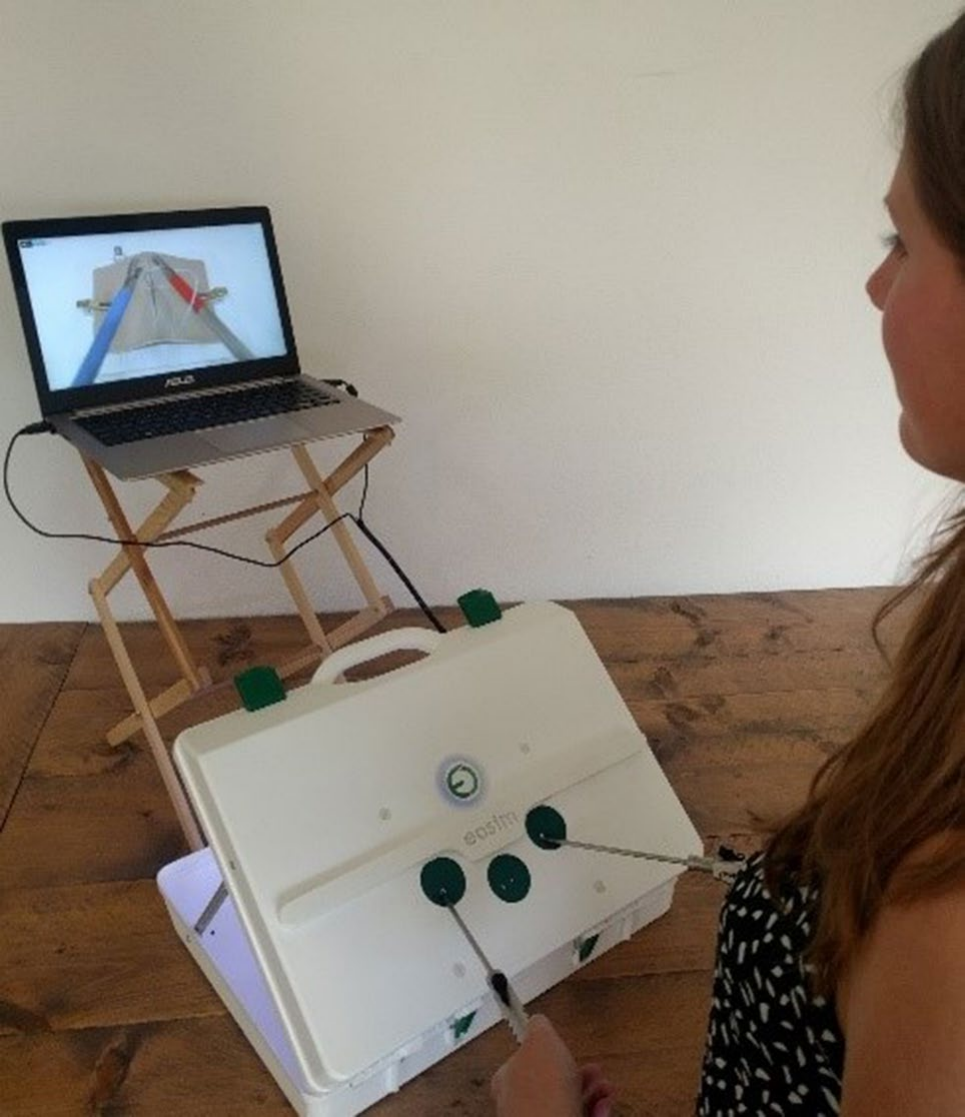
Setup of the eoSim laparoscopic simulator

For the training of the laparoscopic group the eoSim augmented reality simulator was used (Eosurgical ltd., Edinburgh, Scotland, United Kingdom) (Fig. 1). This simulator was selected due to the objective parametric recording without the virtual reality aspect and therefore, maintaining realistic instrument force feedback. The eoSim consists of a validated inanimate box trainer setup with a laptop and supplied software, to guide the trainee and track the instrument tips (Augmented Reality) [14–17]. During training the standard supplied needle holders were used for all three tasks. The working height of the eoSim was adjusted for each participant and adjustable laptop standards were constructed to allow the screens to be kept at eye level. The supplied Surgtrac software allowed for instrument tracking by colored markings at the tip of the instruments (blue for the left hand and red for the right hand). The parameters tracked by the software were ‘time’ ‘distance’, ‘working space’ (average distance between instruments) and ‘off-screen’ as specified in Table 1. Additional outcomes such as the adequate knot and deviation from the marked target were measured after completion of each task by the researcher.

**Table 1:** Parameter definitions

General parameters	Definition
Total time	Total time when the user begins the task and when the user finishes or exits the exercise in seconds
Total distance	Distance travelled by all instruments in meter
Adequate knot	Was a surgical knot created by placing a double wrap followed by two counter wise single wraps
Off-screen	Percentage of the time the mentioned instrument was off-screen
Needle precision	Number of needle punctures on relevant target mark/total number of needle punctures.
eoSim parameters	
Working space	The average distance between instruments in square meter
Needle drops	Number of times de needle was dropped
RobotiX parameters	
Number of movements	Total number of instrument movements
Inaccurate punctures	Sum of deviation from each needle puncture to the edge of marked area distance outside of target’s radius (mm).

#### RobotiX robot assisted surgical simulator

**Fig. 2:**
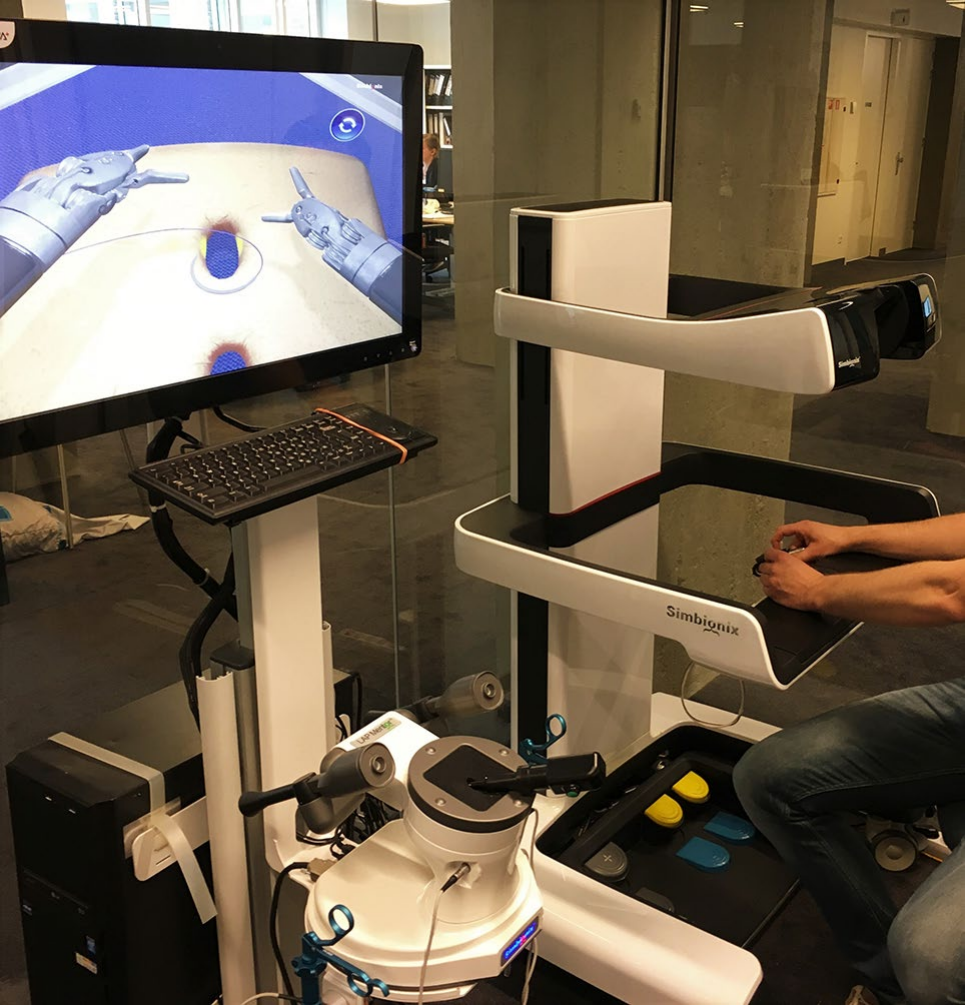
Setup of the RobotiX robot assisted VR simulator

The RobotiX Virtual Reality robot assisted surgery simulator was used for the robot assisted group in a standard supplied setup (3D-systems Inc., Cleveland OH) (Fig. 2) [18–25]. This setup consists of a main console for the trainee and a tower component, containing the supplied computer and task control. The system was able to be adjusted to each participant’s preferences in terms of height and wideness of the 3D viewer and the straps for the master controllers. The supplied software ‘Mentorlearn’ is a web-based simulator curriculum management program which allowed for a user specific account to be created, therefore the curriculum created for this study could be assigned to the participant and kept track of. The system recorded multiple parameters in terms of ‘time’, ‘movement’ and ‘safety’ for each task, which are displayed in Table 1. The RobotiX system was used for this study as the virtual reality system allows for training outside of the operating room and without the use of an expensive Davinci system.

### Tasks

Tasks performed in this study were selected based on the different aspects required during complex suturing. Task 1 consisted of a intracorporeal suturing task which focusses on the suture placement and knot tying. The railroad track (Task 2) was a continuous needle transfer task in a tilted plane. The vaginal cuff closure (Task 3) was a anastomosis needle transfer task by closing the vaginal cuff with a barbed suture. The laparoscopic Task 2 and 3 on the eoSim where developed by the researchers and validated in a previous study [17]. A specific task description is found in the supplemental section of this study.

### Protocol

After recruitment all participants completed the questionnaire regarding their demographics, surgical experience and consent. This was followed by an instruction of either the use of the eoSim or the RobotiX simulator. RobotiX participants were instructed not to use the camera function, as this was not available on the eoSim. Accordingly, two basic introduction tasks were performed to get acquainted with the system after which the repetition of the three suturing tasks could begin. Participants were allowed to train under guidance of a researcher in multiple sessions each of maximal an hour, to prevent fatigue. A researcher recorded the parametric data, number of repetitions and scored the parameters not measured by the eoSim such as the ‘adequate knot’ parameter.

The learning curve for Task 1 was defined to be completed after either performing the task twenty times or scoring three subsequently similar performance scores in a row, representing a plateau phase. The number of repetitions was based on a previous study by Botden et al. where a laparoscopic suturing learning curve was shown within fifteen repetitions [5]. Due to the number of repetitions, visual similarity and outcome comparability Task 1 was used as the ‘Main task’ for the determination of the learning curve. Task 2 was completed by performing three repetitions on the RobotiX (each repetition consisted of five needle transfers). On the eoSim Task 2 was completed by either fifteen repetitions or three consecutive similar performances. The anastomosis needle transfer task was completed on both systems by performing three repetitions.

### Data analysis

Power analysis (*β *= 0.2 and *α *= 0.05) showed a minimal of 16 participants for each group was required to show a difference of 100 s at the end of the suturing learning curve. Data from both systems was extracted and sorted by participants ID number. Data was accordingly combined in a database with the questionnaire results using the Statistical Package for Social Sciences (SPSS) software, version 22 (IBM Corp., Armonk NY). Due to the skewness of the data, non-parametric analyses were used for the calculation of the *p* values. A *p* value of < 0.05 was considered significant. To further analyze the length of a learning curve the cumulative sum analysis (CUSUM) was used. The CUSUM method used in this study has been suggested in other learning curve studies to investigate the phases of learning in the learning curve. This method transforms the raw data into the running total of data deviations from their group mean, enabling researchers to visualize the data for trends not discernible with other approaches. An important advantage of the CUSUM analysis is that it is able to identify subtle and slow changes, which was otherwise not visualized in the robot assisted surgery learning curve [10, 11]. Finally, a sub-analysis between the novice and laparoscopic experienced robot assisted participants to determine the influence of laparoscopic experience on the robot assisted learning curve outcomes.

## Results

### Demographics

This study enrolled a total of 47 participants of which 17 completed the laparoscopic learning curve and 30 the robot assisted. The mean age was 23 years in the laparoscopic and 30 in the robot assisted group. The male–female ratio was 35:65 versus 43:57 and a right handedness percentage of 94% versus 87% between the laparoscopic and robot assisted group respectively. The laparoscopic group consisted of only medical interns as these participants were not allowed to have previous laparoscopic experience. Due to the laparoscopic experience in the robot assisted group, more participants were included in this group to allow for a further sub-analysis to be performed. Therefore, the robot assisted group consists of 13 medical interns, 17 laparoscopic experienced participants of which 13 residents in training and four specialized surgeons, all without any robot assisted experience.

### Learning curve progression

The length of the learning curve of the participants was based on Task 1 in both groups. In the laparoscopic group nine participants performed 20 knots, and all completed at least 14 knots in the laparoscopy group. For the robot assisted group 21 participants completed the full training session and all completed at least nineteen knots. Regarding Task 2, two participants, from the laparoscopic group, were unable to complete the last run (repetitions 11–15) due to limited time availability during the study. Their previous repetitions were included to adhere to an intention to treat analysis and prevent possible bias. A similar procedure was followed for Task 3 in which one laparoscopic participant was unable to complete all three repetitions.

### Task 1 intracorporeal suturing

**Fig. 3:**
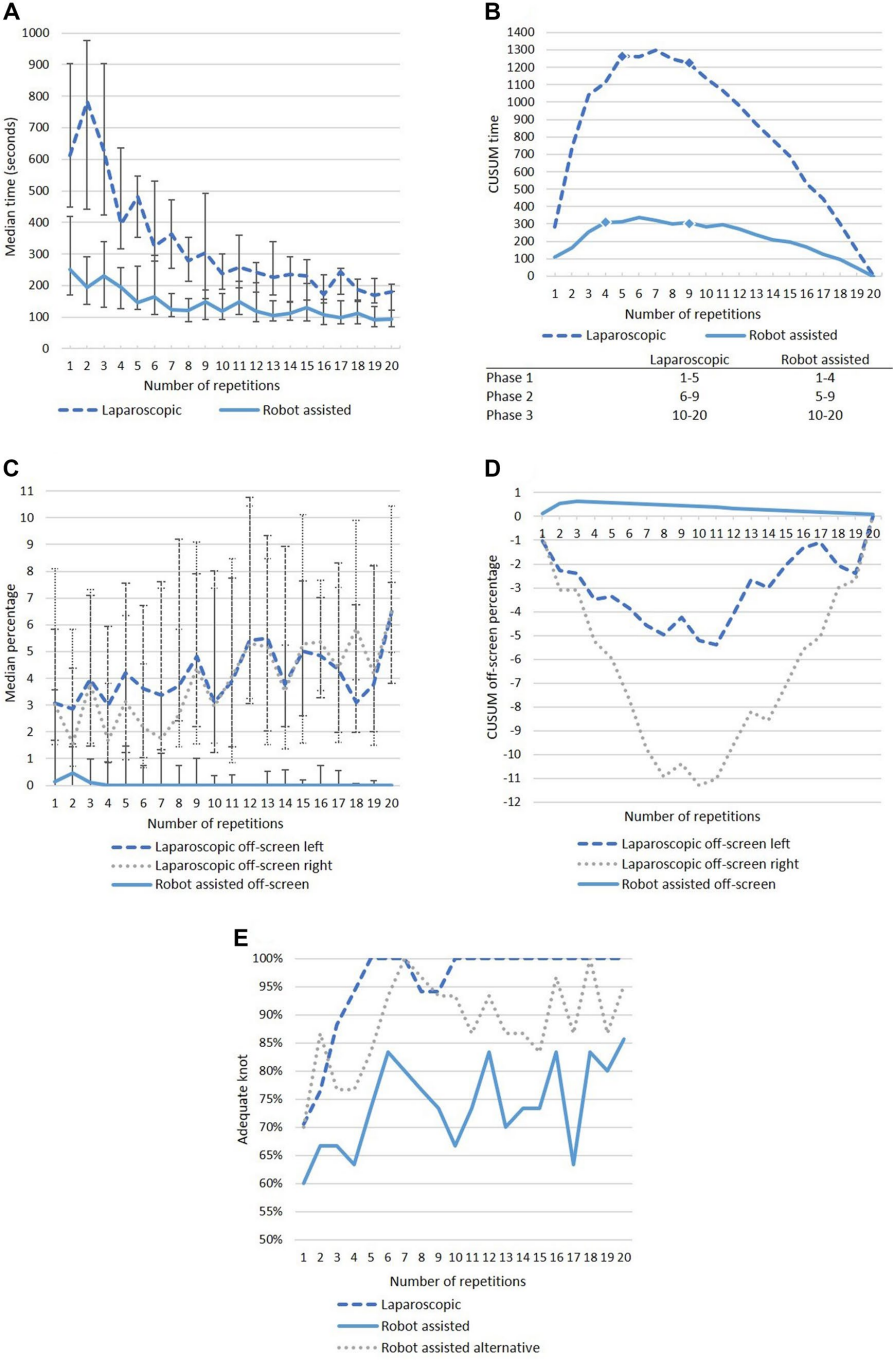
Outcome graphs regarding Task 1 intracorporeal suturing learning curve for the laparoscopic and robot assisted group. **A** Median suturing time in seconds (25–75th interquartile range). **B** Cumulative sum of task time (CUSUM time). **C** Median percentage instruments off-screen (25–75th interquartile range). **D** Cumulative sum instruments off-screen percentage (CUSUM off-screen). **E** Overall percentage adequate knot

Main results of the intracorporeal learning curve are shown in Fig. 3 (A–E). The time median time required to complete Task 1 at the start of the learning curve was 611 s in the laparoscopic group versus 251 s in the robot assisted group (*p *< 0.001). However, the laparoscopic group median time results in a steep curve reducing the time from 611 s at repetition 1 to a median of 186 s at the 18th repetition (70% reduction). For the robot assisted group, the time results in a reduction of 251 s at the first repetition to 112 s at the 18th repetition (55% reduction). The CUSUM time shows a peak at repetition five and nine for the laparoscopic group and at repetition four and nine for the robot assisted group. This shows both groups have a similar characteristic of the learning curve peaks, which can be divided in the following phases: phase 1 (laparoscopic 1–5, robot assisted 1–4), phase 2 (laparoscopic 5–9, robot assisted 4–9) and phase 3 (laparoscopic and robot assisted 10–20). Direct comparison of the laparoscopic versus robot assisted median time results in a significant *p* value of < 0.001 for phase one (592 s vs. 232 s, respectively) two (352 s vs. 159 s, respectively) and three (240 s vs. 128 s respectively). In the robot assisted group, off-screen percentage was 0.4–0% within four repetitions (Fig. 3C). The laparoscopic group shows a trend of increasingly more off-screen percentage for both instruments, although there was a high variability for this parameter between the participants, based on the wide range (Table 2). The CUSUM analysis of the off-screen percentage shows a negative curve for the laparoscopic participants due to the increase of percentage off-screen with a peak at repetition ten (Fig. 3D). The correctness of the performed knot is shown in Fig. 3E. The laparoscopic participants managed to show a steep learning curve and scored 100% within five repetitions. The robot assisted participants started at a lower percentage of 60% which gradually increases to 86% at the 20th repetition. The alternative analysis is shown in which a square knot is also considered an adequate knot to correct for the possible VR bias. This learning curve shows a steeper and higher curve as compared to the initial analysis. This curve starts with 70% and reaches 100% at the seventh repetition.

**Table 2:** Median (25–75th quartile) values of the intracorporeal suturing task (Task 1) per learning curve phase

	Phase 1	Phase 2	Phase 3	*p* values		
	Knot 1–5	Knot 6–9	Knot 10–18	1 vs. 2	1 vs. 3	2 vs. 3
Laparoscopic (*n *= 17)						
Time (s)	591 (468–820)	352 (255–415)	240 (191–272)	**0.001**	**0.000**	**0.000**
Off-screen left (%)	4.4 (2.4–5.6)	4.6 (2.5–8.4)	5.6 (3.2–7.3)	0.068	0.113	0.981
Off-screen right (%)	4.2 (2.9–5.1)	4.0 (1.6–6.5)	6.2 (3.7–7.1)	0.723	**0.007**	**0.025**
Adequate knot (%)*	86 (20)	97 (8)	100 (0)	0.039	**0.009**	0.163

Robot assisted (*n *= 30)	Knot 1–4	Knot 5–9	Knot 10–18	*p* values		
Time (s)	232 (168–309)	159 (127–233)	128 (102–177)	**0.000**	**0.000**	**0.001**
Off-screen total (%)	1.3 (0.1–6.1)	0.7 (0.1–2.2)	0.4 (0.0–0.8)	**0.018**	**0.001**	0.136
Adequate knot (%)*	64 (27)	77 (23)	74 (21)	0.046	0.085	0.483
Adequate knot alternative (%)*	78 (27)	93 (13)	90 (15)	**0.001**	**0.013**	0.388

Data in this table represents median performance scores and 25–75th percentile range. Statistical differences were calculated using non-parametric tests (Wilcoxon)

*Mean values (standard deviation) and paired *T* test were used. *p* values of < 0.05 (displayed in bold) were considered significant

### Task 2 tilted plane needle transfer

The results of the needle transfer are shown in Table 3. The laparoscopic group showed a statistically significant improvement in time between repetition 1–5, 6–10 and 11–15 and the time to complete the task decreased from 2043 s in repetition 1–5 to 557 s in repetition 11–15 (*p *= 0.001). The robot assisted group, however, only showed a statistically significant reduction in time between repetition 1–5 and repetition 11–15 (331 s vs. 307 s respectively, *p *= 0.035). Statistically significant differences were also seen in the laparoscopic group between the first and third run for the parameters ‘working space’ (*p *= 0.031), ‘off-screen right instrument’ (*p *= 0.023), and ‘needle drops’. The ‘total path length’ parameter showed a significant difference between run 1 versus 2 (*p *= 0.001), run 2 versus 3 (*p *= 0.001) and run 1 versus 3 (*p *= 0.036) with a 78% percent reduction in instrument meters. The robot assisted group did not show significant improvements in either of the remaining parameters.

**Table 3:** Median (25–75th quartile) values of the tilted needle transfer task (Task 2) per run (five sutures in each run)

				*p* values		
	Run 1: 1–5	Run 2: 6–10	Run 3: 11–15	1 vs. 2	1 vs. 3	2 vs. 3
Laparoscopic (*n *= 17)						
Time (s)	2043	1119	557	**0.019**	**0.001**	**0.012**
	(1508–3065)	(787–2193)	(390–1057)			
Working space (m^2^)	5.9	2.9	3.1	**0.049**	**0.031**	0.798
	(3.2–7.8)	(1.0–6.9)	(1.5–5.0)			
Off-screen left (%)	32	68	61	0.055	0.191	0.865
	(21–56)	(22–87)	(23–77)			
Off-screen right (%)	23	29	28	0.227	**0.023**	0.112
	(18–31)	(14–41)	(21–40)			
Needle precision (%)	80	100	100	0.233	0.101	1.00
	(60–100)	(80–100)	(80–100)			
Needle drops	2	1	0	0.140	**0.003**	0.065
	(0.5–3.0)	(0.0–2.5)	(0.0–1.0)			
Total path length (m)	64	34	14	**0.001**	**0.001**	**0.036**
	(40–94)	(16–45)	(7–34)			
Robot assisted (*n *= 30)						
Time (s)	331	306	307	0.289	**0.035**	0.558
	(253–449)	(226–376)	(234–366)			
Number of movements	551	510	507	0.517	0.299	0.572
	(434–646)	(412–604)	(414–600)			
Off-screen total (%)	0.00	0.00	0.00	0.904	0.557	0.715
	(0.00–0.18)	(0.00–0.11)	(0.00–0.21)			
Needle precision (%)	49	44	43	0.754	0.704	0.382
	(31–58)	(32–51)	(36–60)			
Inaccurate punctures	9.5	10.5	8.5	0.532	0.845	0.737
	(5.8–13)	(6–16)	(6–17.5)			
Total path length (m)	5.9	5.5	5.5	0.673	0.666	0.934
	(4.3–6.9)	(4.2–7.1)	(4.1–7.2)			

Data in this table represents median performance scores and 25–75th percentile range. Statistical differences were calculated using non-parametric tests (Wilcoxon). *p* values of < 0.05 (displayed in bold) were considered significant

### Task 3 anastomosis needle transfer

The training of the anastomosis needle transfer task, as shown in Table 4, resulted in a statistically significant difference in the parameter time between the first and third run (laparoscopic 1570 s vs. 833 s, *p *= 0.015 and robot assisted 352 s vs. 261 s, *p *= 0.006) and the second versus third run (laparoscopic 1131 s vs. 833 s, *p *= 0.002 and robot assisted 311 s vs. 261 s, *p *= 0.009). The laparoscopic participants improved their working space statistically significantly between run one and two (4.9 m^2^ vs. 4.5 m^2^, *p *= 0.011). The left hand off-screen percentage however, did increase from 1.7 to 7.4% between run one and two (*p *= 0.044) and from 1.7 to 7.3% (*p *= 0.044) between run one and three. Similar results were found for the right hand off-screen percentage with median from 1.2 to 2.2% and 2.0% respectively, without statistically significant differences. The robot assisted participants did improve their off-screen percentage from 1.1 to 0.7% and 0.9% respectively, although not statistically significant. However, a statistically significant reduction in path length was observed between the second and third run for both the laparoscopic (25 m vs. 13 m, *p *= 0.004) and robot assisted group (7.4 m vs. 6.6 m, *p *= 0.026).

**Table 4:** Median (25–75th quartile) learning curve values of the anastomosis needle transfer task

	Run 1: Stitch 1–8	Run 2: Stitch 9–16	Run 3: Stitch 17–24	*p* values		
				1 vs. 2	1 vs. 3	2 vs. 3
Laparoscopic (*n *= 16)						
Time (s)	1570	1131	833	0.109	**0.015**	**0.002**
	(869–1933)	(963–1284)	(683–992)			
Working space (m2)	4.9	4.5	4.5	**0.011**	**0.024**	0.352
	(4.6–5.9)	(3.5–5.2)	(4.0–5.0)			
Off screen left (%)	1.7	7.4	7.3	**0.044**	**0.044**	0.877
	(0.8–4.2)	(3.9–17.5)	(3.8–11.9)			
Off screen right (%)	1.2	2.2	2.0	0.408	0.074	0.379
	(0.5–2.5)	(0.7–3.8)	(1.6–3.2)			
Needle precision (%)	60	60	63	0.278	0.271	0.421
	(16–75)	(28–84)	(50–88)			
Total path length (m)	25	25	13	0.836	**0.017**	**0.004**
	(13–39)	(20–32)	(10–24)			

Robot assisted (*n *= 30)	Run 1: stitch 1-10	Run 2: stitch 11–20	Run 3: stitch 21–30	*p* values		
Time (s)	352	311	261	0.213	**0.006**	**0.009**
	(267–415)	(253–391)	(203–352)			
Number of movements	609	589	483	0.673	**0.028**	**0.010**
	(484–747)	(485–694)	(409–621)			
Off-screen total (%)	1.1	0.7	0.9	0.552	0.905	0.417
	(0.2–2.8)	(0.2–2.6)	(0.1–4.1)			
Needle precision (%)	89	86	89	0.777	0.550	0.210
	(83–93)	(82–93)	(82–93)			
Total path length (m)	7.9	7.4	6.6	0.902	0.138	**0.026**
	(6.3–8.6)	(6.0–8.9)	(5.7–8.9)			

Data in this table represents median performance scores and 25–75th percentile range. Statistical differences were calculated using non-parametric tests (Wilcoxon). *p* values of < 0.05 (displayed in bold) were considered significant

### Laparoscopic experience sub-analysis

**Fig. 4:**
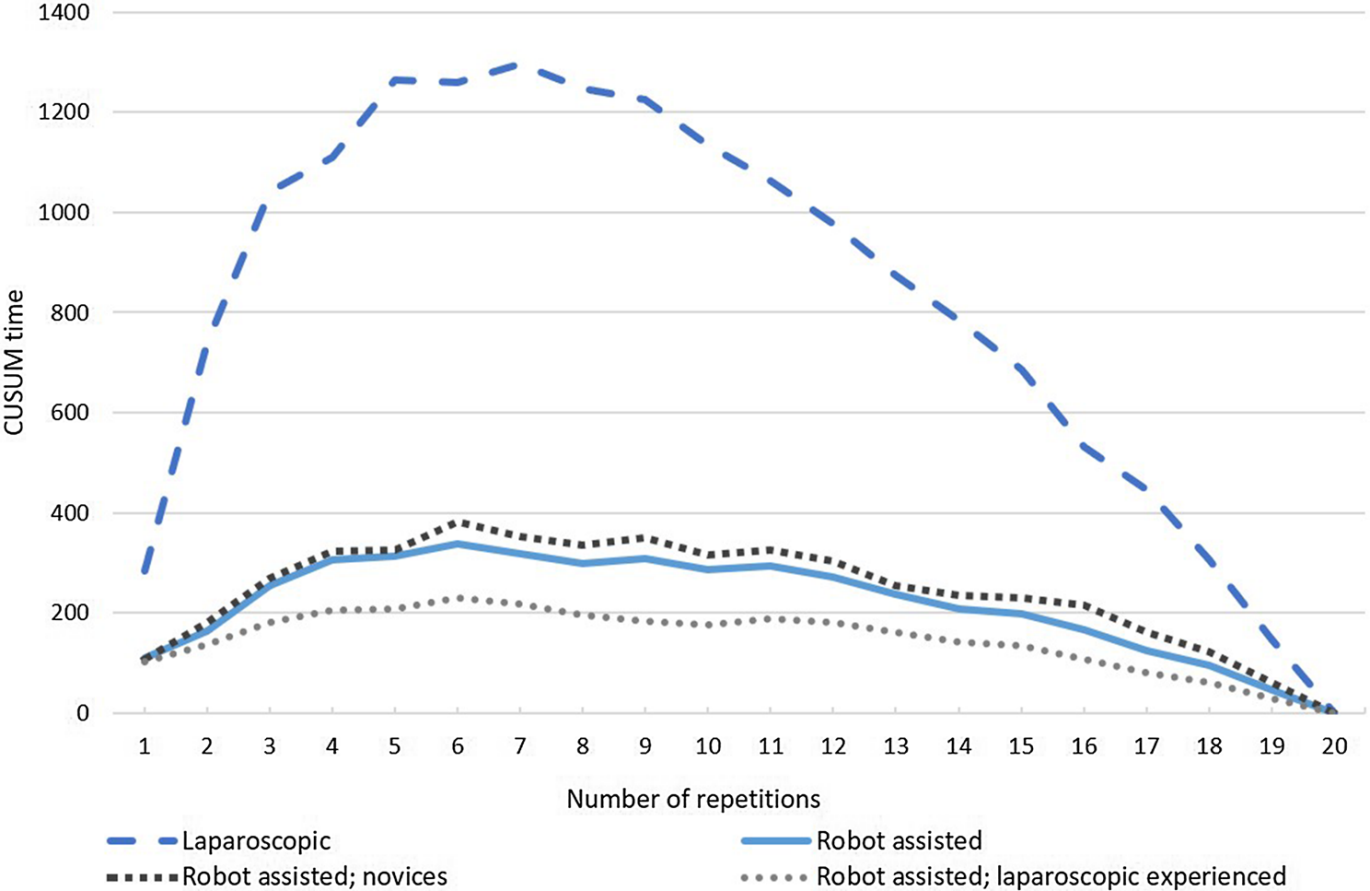
Sub-analysis of cumulative sum of task time (CUSUM time) on the novices and laparoscopic experienced participants in the robot assisted group

The robot assisted group consisted of novices (*n *= 13) and laparoscopic experienced (*n *= 17) participants, and a sub-analysis was performed to identify differences between both the robot assisted group. Statistically significant differences between novices and laparoscopic experienced participants were found for Task 1 in the median overall suture time for phase 1 (median knot 1–4; 300 s vs. 182 s, *p *= 0.039)showing faster initial suturing time in laparoscopic experienced participants. Suturing time at knot 2 (median 228 s vs. 160 s, *p *= 0.039) and knot 16 (median 139 s vs. 97 s, *p *= 0.032) was longer in novices as compared to laparoscopic experienced participants. Subsequently, statistically significant difference was found at the adequate knot alternative parameter at phase 1 (88% vs. 69%, *p *= 0.046) and phase 3 (97% vs. 86%, *p *= 0.029) novices versus laparoscopic experienced respectively. Figure 4 shows the CUSUM time and a more plane curve for the laparoscopic experienced group. Sub-analysis of experience for Task 2 did not result in any statistically significant differences. For Task 3 only the outcome of the needle precision parameter was statistically significant between novices and laparoscopic experienced participants at phase 2 (86% vs. 90%, *p *= 0.026).

## Discussion

The results of this study show that the robot assisted learning curve follows a short completion time at the start with a more horizontal curve compared to the laparoscopic learning curve when focusing on the time to complete the suture. The laparoscopic learning curve however, has a higher suture completion time at the start with a steeper curve than in robot assisted suturing. A ‘steep’ learning curve is often mistaken for a period of learning where the progress is slow, however, it refers to the rapid improvement in outcomes due to learning [2]. This indicates fast learning in laparoscopic complex suturing and more intuitive abilities with less improvement in robot assisted surgery. Additionally, laparoscopic experience is indicated to reduce the initial robot assisted suturing time. Although the differences between the beginning and plateau phase is much higher in laparoscopic suturing, the length and characteristics of the phases are similar, when analyzed with the CUSUM method (Fig. 3B). Based on the large difference in ‘time’ and ‘off-screen’ percentage during the performed tasks, there is an advantage in robotic training of complex minimal invasive suturing regarding these parameters. This was however not shown for the ‘adequate knot’ and ‘needle precision’ parameters.

Previous studies have been performed to evaluate the learning curve, although these mainly focused on clinical settings. The study by Chandra et al. compared the learning curve of laparoscopic and robot assisted simulated suturing for novices using only one simulator modality [1]. Results of this study demonstrated a consistently better time performance for the robot assisted group. The time performance of the robot assisted novices scored a mean time of 215 s for the first three knots (225 s current study) and 193 s for the last two knots (134 s current study) from a total of ten repetitions. The current study resulted in similar learning curve time values (Table 2) as the study by Chandra et al. with substantial better performance for robot assisted surgery compared to laparoscopic surgery. This indicates that despite the use of different simulator modalities (virtual reality and augmented reality) used in the current study, the potential bias on the learning curve results were limited. The better performance outcome in the first phase of the learning curve for the robot assisted group as shown in this study is also corroborated by the studies of Passerotti et al. and Marecik et al. [8, 9], and demonstrates the more intuitive abilities of robot assisted procedures. For further evaluation of the intuitive beneficial effects, it would be interesting to compare the learning curve of complex suturing skills with the use of ‘wristed’ 3D laparoscopic instruments versus robot assisted use of wristed instruments. This to identify the true additive value of robot assistance over the advantage of six degrees of freedom during these complex tasks.

Due to the inclusion of laparoscopic experienced participants in the robot assisted group, the robot assisted learning curve was performed by a larger group as compared to the laparoscopic learning curve. To determine the effect of laparoscopic experience on the robot assisted learning curve a sub-analysis was performed. This resulted in some statistically significant differences. An important finding is that the median time of the first four robot assisted suturing knots by novice participants took significantly longer to complete than four suturing knots by the laparoscopic experienced participants (300 s vs. 182 s, *p *= 0.039). This shows that laparoscopic experience can be beneficial in completion time during the first phase of the learning curve. However, laparoscopic experience appears to have a negative influence on the specific knot alternative parameters. This is shown for Task 1 during phase 1 and 2 since novices outperformed laparoscopic experienced participants (88% vs. 69%, *p *= 0.046 and 97% vs. 86%, *p *= 0.029 respectively).

It is important to notice that time to complete the suture is not the ideal parameter to assess the accuracy of the procedure, and therefore other parameters were evaluated in this study as well. The safety parameter ‘off-screen’ showed a change effect in the laparoscopic group, while the robot assisted group already had a positive outcome on these parameters and this did not change significantly (Fig. 3C–E and Tables 2, 3 and 4). However, the off-screen percentage for the laparoscopic group actually increased during repetition of all three tasks. This could be based on the fact that the RobotiX focusses graphically on this aspect in the result screen after the run of the task and also gives an error sign during the procedure. The high off-screen percentages found for the laparoscopic group at Task 2 can possibly be explained by the task mechanics. Due to the tilted plane the working area was close to the camera which resulted in a small working space and more percentage instruments off-screen. The difference between the right and left off-screen percentage can be explained by the right handedness of most participants. It is, however, an important factor to limit off-screen time because of the potential collateral damage to surrounding organs, particularly for still inexperienced residents or surgeons. When evaluating the knot accuracy (Fig. 3E), a dip in the learning curve at repetition eight and nine for the laparoscopic group and between repetition eight and fifteen for the robot assisted group was found. This phenomenon is seen more often in learning curves and could be based on fatigue, frustration or more focus on a faster time than on the perfect knot [5]. A previous study also showed that the frustration level was lower when practicing suturing skills in a robotic assisted simulator than a laparoscopic simulator, indicating that this fact could be beneficial in the learning process as well [9].

### Strengths and limitations

The main strength of this study is the randomization of the novice group, the relative high number of participants and the inclusion of multiple assessment parameters. This study was also aimed at using multiple assessment parameters for proficiency as stated by the recent review from Kassite et al. [10]. These parameters are found in the correctness of the knot and precision of the needle punctures; the safety parameter ‘out of view’ which indicates the risk on collateral damage and therefore, is an indicator for complications; and time to finish the procedure.

Due to the differences in simulator modalities, a direct comparison of outcomes was not possible. In an ideal situation a comparison on the same simulator would be preferred to directly compare outcome parameters. Besides the simulator itself, the outcome parameters were also difficult to compare, because the parameters on the VR system were not adjustable. In addition, a limitation of the predefined tasks from a VR simulator was the inability to adjust tasks to make them more similar to the laparoscopic simulator, therefore the laparoscopic tasks were adapted to match the robot assisted tasks. Another limitation of VR simulation was observed during the suturing task with the simulated thread. During the tying of the knot the system response was not always correct concerning the tied knot by confusing a double wrap with a single wrap for example. This led to a high variation of the correct knot parameter of the robot assisted group and influenced their result negatively. This phenomenon was also shown in previous studies on VR simulators, but could not be adverted in this study, because there were no non-VR robotic trainers available [26].

Since surgeons consider surgical knot and multiple square knots as solid, we performed a second analysis of this parameter, also including the square knots in the robot assisted group (Fig. 3E). This second analysis shows more similarities to the laparoscopic curve, correcting for the possible VR bias. In addition, the laparoscopic correctness of the knot was tested on the strength of the knot, but not by an objective assessment form, therefore not confirming the method of knot tying.

## Conclusion

This study shows that robot assisted complex suturing shows a horizontal learning curve, in which there is little learning effect, because the outcomes in the simulator are good from the start. The learning curve of the laparoscopic training is steep and especially time to complete the suture is significantly slower in all phases of the suturing learning curve as well as the tilted plane and anastomosis needle transfer task compared to the robot assisted group. Therefore, the use of robotic assistance could be most beneficial for novice surgeons performing complex suturing tasks and thereby avoiding the possible additional learning curve associated morbidity when otherwise performed laparoscopically.

## Electronic supplementary material

Below is the link to the electronic supplementary material.

Supplementary material 1 (JPEG 102 kb)

Supplementary material 2 (JPEG 146 kb)

Supplementary material 3 (JPEG 112 kb)

Supplementary material 4 (JPEG 92 kb)

Supplementary material 5 (JPEG 92 kb)

Supplementary material 6 (JPEG 132 kb)

Supplementary material 7 (DOCX 22 kb)
