# Supplementary figures and images for: Robot assisted versus laparoscopic suturing learning curve in a simulated setting
Source: Surg Endosc. 2019 Nov 21;34(8):3679–89. doi: 10.1007/s00464-019-07263-2 (PMC7326898; doi:10.1007/s00464-019-07263-2)

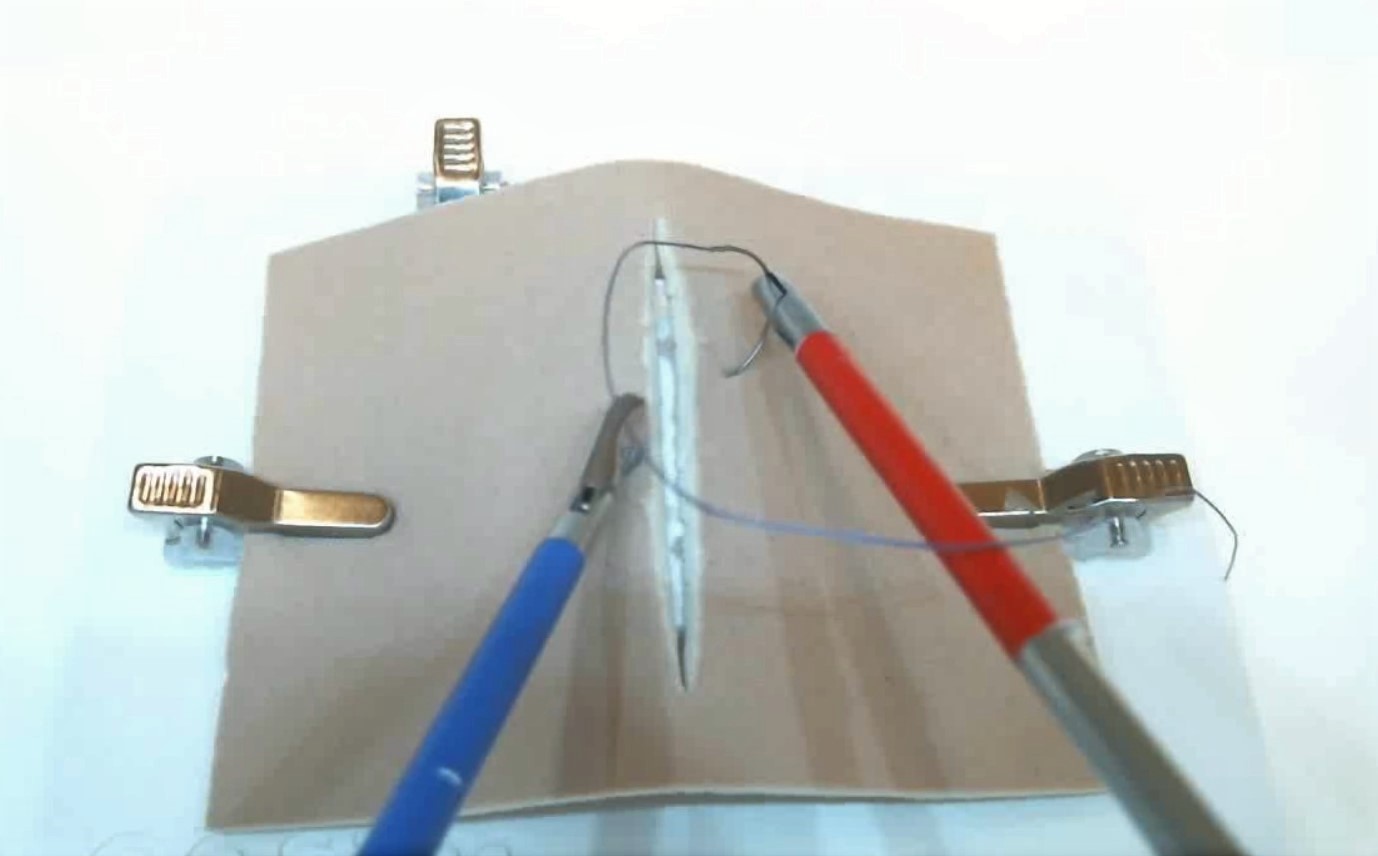

Supplement: Supplementary file 1 — Supplementary material 1 (JPEG 102 kb) [file 464_2019_7263_MOESM1_ESM.jpg]

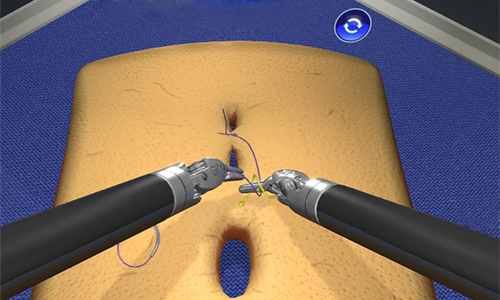

Supplement: Supplementary file 2 — Supplementary material 2 (JPEG 146 kb) [file 464_2019_7263_MOESM2_ESM.jpg]

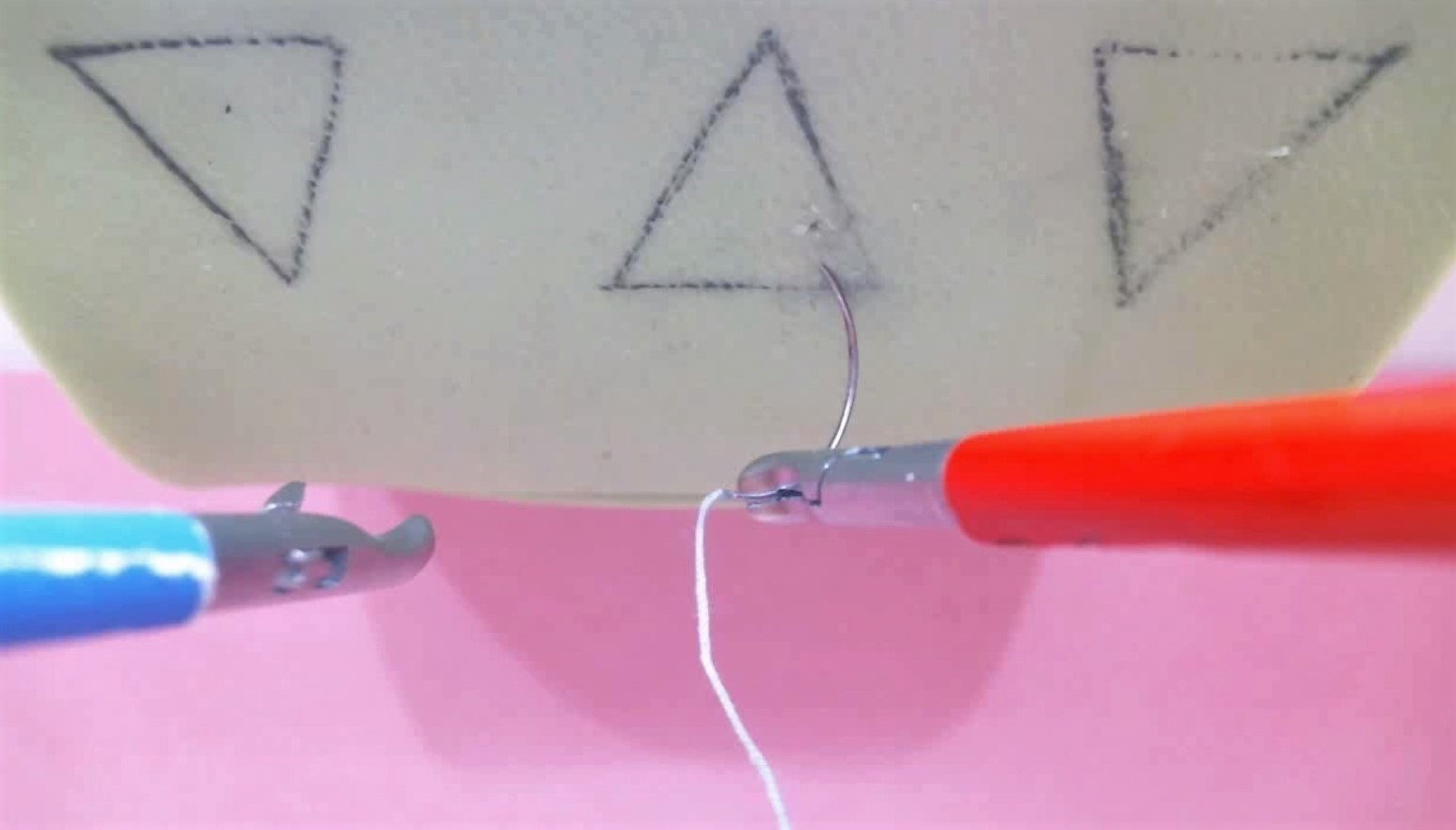

Supplement: Supplementary file 3 — Supplementary material 3 (JPEG 112 kb) [file 464_2019_7263_MOESM3_ESM.jpg]

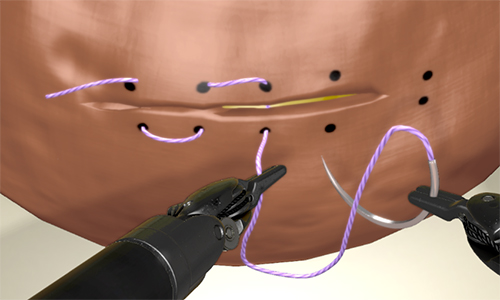

Supplement: Supplementary file 4 — Supplementary material 4 (JPEG 92 kb) [file 464_2019_7263_MOESM4_ESM.jpg]

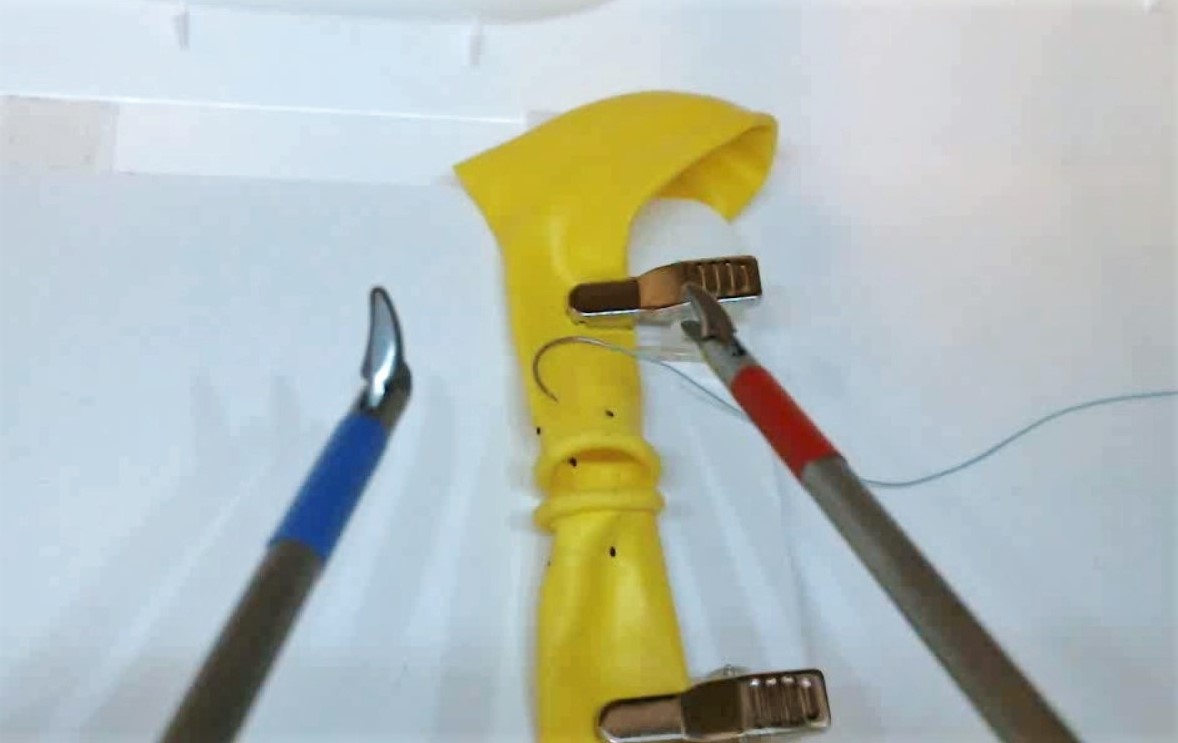

Supplement: Supplementary file 5 — Supplementary material 5 (JPEG 92 kb) [file 464_2019_7263_MOESM5_ESM.jpg]

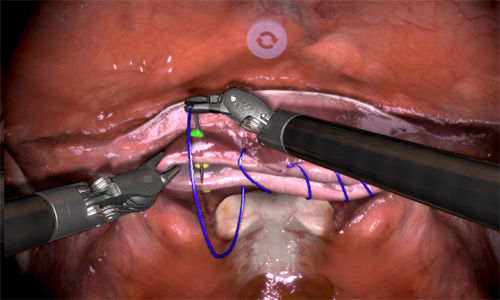

Supplement: Supplementary file 6 — Supplementary material 6 (JPEG 132 kb) [file 464_2019_7263_MOESM6_ESM.jpg]
