# Supplementary material for: Robot assisted versus laparoscopic suturing learning curve in a simulated setting
Source: Surg Endosc. 2019 Nov 21;34(8):3679–89. doi: 10.1007/s00464-019-07263-2 (PMC7326898; doi:10.1007/s00464-019-07263-2)
Supplement: Supplementary file 7 — Supplementary material 7 (DOCX 22 kb) [file 464_2019_7263_MOESM7_ESM.docx]

Supplemental 1: Task descriptions

Task 1: Intracorporeal suturing and knot tying: this task consisted of a single suture to be placed on a suturing pad with the appropriate surgical knot (double wrap followed by two counter clockwise single wraps). Laparoscopic participants performed this task similar to the robot assisted participants, both with a suture pad in the horizontal plane, containing a vertical defect (Figures S1 and S2). In both the laparoscopic and robot assisted group two needle drivers were used to complete the task. For the laparoscopic group a standard twenty-centimeter braided suture with a 30mm curved needle was used. The suture in the RobotiX was standard in the VR program. The task was completed when the second single wrap was placed and the suture was ready to be cut. This task was taken for the main visualization of the learning curves of the participants, due to the equality between the two simulators and repetitive nature of the knots.

Figure S1 and S2: Task 1 intracorporeal suturing on the eoSim (Figure S1) and RobotiX (Figure S2)

Task 2: Tilted plane needle transfer: the laparoscopic participants performed this task on an 80-degree tilted plane where a needle was required to enter and exit within the marked areas (Figure S3 and S4). The robot assisted participants performed this task on a virtual suturing pad with an approximate 90-degree tilted plane (Figure S4). The task consisted of a needle to be transferred in a mattress pattern through all the highlighted dots to complete the task. In both groups two needles drivers were used during the task. The laparoscopic repetitions were individually recorded and accordingly summed up per five punctures. The robot assisted tasks consisted of five punctures to complete one task repetition. The tasks were completed three times during the learning curve training for each group.

Figure S3 and S4: Task 2 tilted plane needle transfer on the eoSim (Figure S3) and RobotiX (Figure S4)

Task 3: Anastomosis needle transfer: this task was performed on the eoSim by placing eight sutures without knot tying between two balloon ends, each with four marked dots (Figure S5). For the laparoscopic group a standard twenty-centimeter braided suture with a 30mm curved needle was used. The anastomosis task on the RobotiX consisted of the simulated “vaginal cuff closure” (Figure S6. This task was performed by placing ten sutures with a barbed thread which had to be transferred through five marked dots on each side of the simulated defect. The suture in the RobotiX was standard in the VR program. In both groups two needle drivers were used during the task. The tasks were completed three times during the learning curve training for each group.

Figure S5 and S6: Task 3 anastomosis needle tranfer on the eoSim (Figure S5) and RobotiX (Figure S6)
